# Combined Dietary *Spirulina platensis* and *Citrus limon* Essential Oil Enhances the Growth, Immunity, Antioxidant Capacity and Intestinal Health of Nile Tilapia

**DOI:** 10.3390/vetsci11100474

**Published:** 2024-10-04

**Authors:** Safaa E. Abdo, Abeer F. El-Nahas, Rabab E. Abdellatif, Radi Mohamed, Mohamed A. Helal, Mahmoud M. Azzam, Alessandro Di Cerbo, Seham El-Kassas

**Affiliations:** 1 Genetics and Genetic Engineering, Department of Animal Wealth Development, Faculty of Veterinary Medicine, Kafrelsheikh University, Kafrelsheikh 33516, Egypt; safaa_abdo2010@vet.kfs.edu.eg (S.E.A.); rabababdellatif12@gmail.com (R.E.A.); 2 Department of Animal Husbandry and Animal Wealth Development-Genetics, Faculty of Veterinary Medicine, Alexandria University, Alexandria 22758, Egypt; 3 Department of Aquaculture, Faculty of Aquatic and Fisheries Sciences, Kafrelsheikh University, Kafrelsheikh 33516, Egypt; radimohamed45@yahoo.com; 4 Animal, Poultry and Fish Breeding and Production, Department of Animal Wealth Development, Faculty of Veterinary Medicine, Kafrelsheikh University, Kafrelsheikh 33516, Egypt; mohamedhelal@vet.kfs.edu.eg (M.A.H.); seham.elkassas@vet.kfs.edu.eg (S.E.-K.); 5 Department of Animal Production, College of Food and Agricultural Sciences, King Saud University, Riyadh 11451, Saudi Arabia; mazzam@ksu.edu.sa; 6 School of Biosciences and Veterinary Medicine, University of Camerino, 62024 Matelica, Italy

**Keywords:** *Spirulina platensis*, lemon peel essential oil, immunity, growth, antioxidant, fat metabolism genes

## Abstract

**Simple Summary:**

Microalgae such as *Spirulina platensis* and essential oils derived from *Citrus limon* have potential growth-promoting, antimicrobial, antioxidant, and immunostimulant effects for several fish species. This manuscript spotlights *Spirulina platensis* and *Citrus limon* essential oil and their roles in improving the performance and health of Nile tilapia. Our results indicate that *Spirulina platensis* and/or lemon essential oil can be used as natural feed additives during aquafeed formulation to improve fish welfare through nutritional management.

**Abstract:**

The dietary presence of feed additives is crucial for boosting fish growth and immunity. Accordingly, this feeding trial aimed to investigate the effects of the separate and concurrent dietary supplementation of *Spirulina platensis* (SP) and bitter lemon (*Citrus limon*) peel essential oil (LEO) on the growth, immunity, antioxidant capacity, and intestinal health of Nile tilapia (*Oreochromis niloticus*). Four groups of male Nile tilapia were employed. The first group (control) was given the basal diet, while the second and third groups received the basal diet supplemented with LEO extract (1%) and SP (1 g/kg diet), respectively. The fourth group received the basal diet supplemented with a mix of LEO (1%) and SP at 1 g/kg. After two months of feeding, using LEO or/and SP improved the overall growth and immunological parameters, with their combination yielding the best outcomes. The supplementation of LEO or/and SP improved the Nile tilapia’s growth metrics and transcriptomic levels of growth-regulating genes such as (*oligo-peptide transporter 1* (*Pep1*), *growth hormone receptors 1* (*GHR1*), and *insulin-like growth factor* (*IGF1*). The improved growth performance was linked to significant increases in the expression levels of mucin and fat metabolism-related genes. Moreover, fish supplemented with LEO, SP, or their combination showed enhanced non-specific immunological measures, including phagocytic and lysozyme activities and the mRNA copies of its regulating genes. Additionally, remarkable increases in the antioxidant enzyme activities and the mRNA levels of their related genes were detected. The *complement* (*C3*) gene’s transcriptomic level was also significantly increased. Furthermore, the dietary supplementation of LEO, SP, or their combination improved the histological structures of the spleen, hepatopancreas, and intestine. The enhanced effects of LEO, SP, or their combination on fish immunity and growth are suggested to be due to their contents of bioactive compounds with anti-inflammatory, antioxidant, and antimicrobial properties. Thus, using the LOE and SP blends as feed additives is recommended for better growth and immunity of Nile tilapia.

## 1. Introduction

Feed additives provide cheap, healthy, eco-friendly alternatives to antibiotics, chemical immune stimulants, and growth promoters [1]. Feed additives include prebiotics, probiotics, phytogenics, or a mix of two types [1]. Most of these additives are rich in bioactive elements, which have a wide range of biological activities such as growth-enhancing, immune-stimulating, antioxidant, and antimicrobial activities [2,3,4]. Recently, the application of several phytogenic products in aquaculture has been recommended as safe and highly effective growth and immune stimulants [5,6,7], with plant-derived essential oils (OS) being used as effective nutritional feed additives in animal and aquaculture sectors [8].

D-limonene is the main component in the citrus that naturally occurs in the peel of citrus fruits, such as oranges or lemons [9]. It is listed, by federal regulations, as generally recognized as safe (GRAS), with a very low toxicity [10]. Limonene has well-known immune-stimulant, anti-inflammatory, and antioxidant impacts in humans and animals [11]. Dietary supplementation of lemon essential oil extracts (LEOs) could improve fish growth performance and intestinal health. Additionally, Nile tilapia fed limonene supplements showed higher weight gains, enhancing *hepatic Insulin growth factor-I* (*igf-1*) gene expression [12].

Moreover, it improved intestinal health and nutrient utilization, confirmed by increasing the villi length and goblet cell number and upregulating the expression levels of intestinal health-related genes [12,13]. Additionally, limonene enhances fish’s antioxidant capacity by increasing the mRNA levels of *catalase* (*CAT*) and *superoxide dismutase* (*SOD*), along with reinforcing the phagocytic and lysozyme activities [14], which protect the cells from toxins and lipid peroxidation (malondialdehyde formation) [15,16]. The limonene in citrus fruit also has strong antibacterial activities against several pathogenic bacterial species, such as *Campylobacter jejuni* and *Staphylococcus aureus* [17,18,19].

Microalgae such as *Spirulina platensis* (SP) are another feed additive that have been recently used in the aquaculture sector due to their highly precious nutritional value [20]. SP has received significant attention in aquaculture as a valuable alternative source of protein instead of those of animal origin [21]. Spirulina protein matches the good-quality reference protein recommended by FAO, as it has a high protein content (70%) in its dry weight and is rich in essential amino acids [22,23,24]. SP dietary supplementation improves the quality of meat fat, as it is richer in polyunsaturated fatty acids than monounsaturated fatty acids [25,26]. Also, dietary supplementation of SP improved many fish species’ growth performance and health status [27,28]. For Nile tilapia, SP improved the growth rate, feed conversion, feed utilization, digestive enzymes, and intestinal health [29,30,31]. It has a strong antioxidant action because of its precious contents of β-carotene, phycocyanin, and tocopherols [32,33,34]. Moreover, it has an optimal level of immune stimulants, minerals, and vitamins, which boost white blood cells (WBCs), phagocytosis, and lysozyme activity [35,36]. SP also improves fish health and water quality by adsorbing heavy metals and nitrites.

The separate dietary supplementation of LEO or SP has been extensively studied, but their synergetic effects, to our knowledge, have yet to be explored. Thus, in this study, we hypothesized that the combined supplementation of both LEO and SP could be more economically effective and induce more effects on Nile tilapia growth performance and immunity. Therefore, this study aimed to evaluate the effects of separate and concurrent dietary supplementation of LEO and SP on Nile tilapia’s growth performance, immune and antioxidant responses, and the transcriptomic profile of growth-, immunity-, antioxidant-, intestinal health-, and fat metabolism-regulating genes.

## 2. Materials and Methods

### 2.1. Ethics Committee Approval

This experimental study was approved by the Institutional Animal Care and Use Committee (IACUC), Kafrelsheikh University, Egypt (Approval number KFS-IACUC/112/2023). All methods were carried out according to the relevant guidelines and regulations of the KFS-IACUC. This study was conducted according to ARRIVE guidelines.

### 2.2. Fish Management

A total of 120 healthy mono-sex (male) Nile tilapia (*Oreochromis niloticus*) fingerlings (average weight 8.00 ± 0.17 g) were collected from a local farm in Kafrelsheikh governorate, Egypt. The fish were kept in glass aquaria (45 cm width × 55 cm length × 23.5 cm height), previously filled with dechlorinated tap water, and half of the water was exchanged daily. The aquaria were supplied with aeration and mechanical filters to remove waste. The water temperature was maintained at 24.7 ± 2.1 °C and pH 7.7–8.6. The fish were kept for adaptation for two weeks, feeding them a commercial tilapia diet (Table 1). Feeding was performed twice daily at a rate of 3% of the fish’s body weight.

### 2.3. Feeding and Experimental Design

The fish were randomly allocated into four groups with three replicates each (10 fish per replicate in each aquarium). The first group was the control group, which was kept on the basal diet (floating feed produced by Aller-Aqua Company, Giza, Egypt) until the end of the experiment. The chemical composition of the basal diet is shown in Table 1. The second group received the basal diet (BD) supplemented with lemon essential oil (LEO) extracts at a 1% concentration, according to Mohamed et al. [13]. The third group was fed BD supplemented with SP at 1 g/kg, according to Al-Deriny et al. [29]. The fourth group was fed BD containing a combination of LEO (1%) and SP at 1 g/kg. The feeding trials continued for two months.

Spirulina and/or LEO were thoroughly mixed with sunflower oil (20 mL/kg diet) and then gently mixed with the feed pellets. The control group’s diet was combined with the same amount of sunflower oil. Finally, the feeds were sealed in vacuum-packed bags and placed into a freezer (−20 °C).

### 2.4. Growth Performance

At the end of the experimental period, the fish were harvested using a suitable net and anesthetized using clove oil (Merck, Darmstadt, Germany) at 50 μL per liter of water [13]. The fish were weighed individually to obtain the final weight of each fish. The fish growth performance and feed utilization were calculated according to El-Kassas et al. [5]. The body weight gain, specific growth rate, feed conversion ratio, and hepato-somatic index were calculated using the following equations:

Body weight gain (BWG) = final body weight (W1)/g − initial body weight (W0)/g

Specific growth rate (SGR %/day) = 100 × (lnW1 − lnW0)/t

Feed conversion ratio (FCR) = feed intake (g)/BWG (g)

Hepato-somatic index (HSI) = 100 × (liver weight/W1)

### 2.5. Sampling

At the end of the feeding trial, blood samples were collected from 6 fish/treatment (2 fish/replicate). Two blood samples were collected from the caudal vein of each fish. One sample was collected on heparin as an anticoagulant for hematological analysis. Another blood sample was collected without an anticoagulant for serum separation. Liver and fore-intestine tissue (proximal part) samples were collected on liquid nitrogen and kept at −80 °C for RNA extraction. Other specimens were collected from the intestine (anterior, middle, and posterior segments), liver, and spleen in Bouin’s solution for histomorphological examination.

### 2.6. Hematological and Immunological Parameter Analysis

Diluted blood samples (with Natt and Herrick’s solution) were used to determine white blood cell (WBC) and red blood cell (RBC) counts. The hemoglobin concentration was measured using the cyanomethemoglobin method using Drabkin’s solution. The microhematocrit method was used to determine the packed cell volume (PCV). For the differential leucocyte count, a blood film for each sample was examined using a computer-assisted light microscope with a 100× oil immersion lens [37].

The phagocytic activity (PA) of the leucocytes to *Candida albicans* was measured in the heparinized blood samples following the methods of Kawahara et al. [38]. The PA was assessed as the percentage of phagocytic cells that engulfed the yeast cells, while the phagocytic index (PI) was calculated by dividing the total number of phagocytized yeast cells by the number of phagocytic cells. Serum lysozyme activity was analyzed using the method described by Abo-Al-Ela et al. [39]. Measurements of the serum lysozyme activity (LZM) depended on comparing the ability of the lysozyme to digest the bacteria cells (*Micrococcus lysodeikticus*) present in 1% agarose gel, giving a clear lysed zone against the lysed zone in a standard plate containing a hen egg-white lysozyme solution of 20 mg/mL.

### 2.7. Serum Biochemical Measurements

The serum total protein and albumin levels were measured using kits from Bio-Diagnostic Co., Dokki, Giza, Egypt, at wavelengths of 550 nm and 630 nm, respectively, according to Doumas et al. [40]. Alanine aminotransferase (ALT) and aspartate aminotransferase (AST) activities were measured at a 540 nm wavelength [41]. Serum cholesterol (CHO) and triglycerides (TGs) were measured using kits from Bio-Diagnostic Co. according to the manufacturer’s instructions. Glucose levels were determined using commercially available kits, according to Ozdemir et al. [42].

### 2.8. Serum Antioxidant Enzyme Activity and MDA Concentration Assessment

The activities of the antioxidant enzymes superoxide dismutase (SOD) and glutathione peroxidase (GPX) and the malondialdehyde (MDA) concentrations were assessed using specific kits (Biodiagnostic, Co., Dokki, Giza, Egypt). The activity of each enzyme was measured using a UV–vis spectrophotometer at a particular wavelength [43,44,45].

### 2.9. Histomorphological Features of Intestine, Liver, and Spleen

The specimens collected from the intestine (anterior, middle, and posterior segments), liver, and spleen were cut into pieces of approximately 0.5 cm^3^ and fixed in Bouin’s solution for 18–24 h. Then, the fixed samples were dehydrated in ascending grades of alcohol, cleared with xylene, and embedded in paraffin wax. Then, 5 μm thick sections were obtained using a rotatory microtome (Leica Rotary Microtome, RM 2145, Leica Microsystems, Wetzlar, Germany) and stained with hematoxylin and eosin stain for histological investigation according to Suvarna et al. [46].

### 2.10. Total RNA Extraction and cDNA Synthesis

The total RNA was extracted from liver and intestinal tissues. Accordingly, a fixed weight of about 50 mg of liver tissue samples was homogenized in phosphate-buffered saline (PBS) and used for total RNA extraction. PBS was used to facilitate the mechanical tissue disruption [47]. For RNA extraction, an intestinal specimen was first ground in a sterile mortar with liquid nitrogen; then, the total RNA was extracted using Trizol (Applied Biotechnology, Giza, Egypt) according to the manufacturer’s manual. The quality of isolated RNA was assessed using 2% ethidium bromide-stained agarose gel electrophoresis. The RNA quantity was analyzed using nanodrop. Two micrograms of the RNA were used for the complementary DNA (cDNA) synthesis using a Thermo-Scientific-Revert-Aid—Frist strand cDNA Synthesis Kit.

### 2.11. Relative Gene Expression Using qPCR

Real-time PCR (qPCR) was used to evaluate the relative expression levels of growth-related genes [*Growth hormone receptors 1* (*Ghr1*) and *Insulin-like growth factor 1* (*Igf-1*)], fat metabolism-related genes [*Fatty acid synthesis* (*Fas*), *Lipoprotein lipase* (*Lpl*), *Fatty acid transport*, *fatty acid binding protein 3* (*Fabp3*)], and cluster of differentiation 36 (*CD36*). The antioxidants *Superoxide dismutase* (*Sod*) and *Catalase* (*Cat*) and innate immune response-related genes *Lysozyme* (*Lzm*) and *Complement* (*C3*) were also evaluated in the liver tissue. Conversely, the relative expression of nutrient absorption and the transporter genes *Mucin-like protein* (*Muc*) and *Oligo-peptide transporter 1* (*Pept1*) were analyzed in the intestine. The data were normalized against two housekeeping genes, *Beta-actin* (*β-actin*) and *Elongation factor-1α* (*Ef-1α*). The preparation of the reaction mixture and the conditions were performed according to Abdo et al. [4]. The specific annealing temperatures and primer sequences for each gene are listed in Table 2. The relative expression levels as fold-changes were calculated based on the 2^−ΔΔCt^ according to the method of Livak and Schmittgen [48].

### 2.12. Statistical Analysis

All data are presented as means ± SEM. A one-way Analysis of Variance (ANOVA) with Duncan’s multiple comparisons test was used to analyze differences among treatments and the control group. All statistical analyses were performed using GraphPad Prism 9.0 (GraphPad^®^ Software Inc., San Diego, CA, USA). A *p* < 0.05 was considered significant.

## 3. Results

### 3.1. Dietary Supplementation of LEO, SP, and Their Mixture Significantly Modified the Growth Performance of Nile Tilapia

The dietary supplementation of LEO, SP, and their mixture significantly increased the final body weights of Nile tilapia compared to the CG (*p* < 0.05) (Table 3).

The effects of the mixture were non-significantly better than those of the separate administration of LEO and SP. The improved final body weights were linked with apparent increases in the body gains and the feed intake, which decreased the FCR (*p* < 0.05). Moreover, the fish’s body length and SGR displayed marked increases with the dietary supplementation of LEO, SP, and their mixture compared to the control (*p* < 0.05). However, the weights of the internal organs, liver, intestine, and HSI were not altered by the dietary administration of LEO, SP, or their mixture.

### 3.2. LEO, SP, and Their Combination Improved the Antioxidant and Non-Specific Immune Responses

Table 4 shows the effects of the dietary supplementation of LEO, SP, and their mixture on antioxidant enzyme concentration, phagocytic activity, index, and lysozyme activity.

Dietary supplementation of LEO, SP, and their mixture to the Nile tilapia’s diet significantly increased the SOD concentration compared to the basal diet, with the highest concentrations found in the case of the SP and LEO + SP mixture (*p* < 0.05). Similarly, dietary supplementation of LEO, SP, and their mixture caused marked increases in GPx concentration compared to the basal diet (*p* < 0.05). The dietary combination of both LEO and SP exhibited the highest GPx concentration compared to the control group and the LEO or SP alone (*p* < 0.05). Increasing the SOD and GPX levels because of LEO, SP, and their mixture was associated with a significant reduction in MDA concentration (*p* < 0.05).

Moreover, the dietary addition of LEO, SP, and their mixture distinctly altered PA, with the highest activities reported in the case of both the LEO and SP mixture, followed by LEO alone compared to the basal diet and SP (*p* < 0.05). LZM activity was also influenced by the dietary addition of LEO, SP, and their combination. The highest activities were noticed in the case of the LEO/SP mixture and LEO alone, followed by SP compared to a non-supplemented basal diet (*p* < 0.05).

### 3.3. The Effects of LEO, SP, and Their Combination on Nile Tilapia Hematological Response and the Serum Biochemical Constituents

Dietary supplementation of LEO, SP, and their combination significantly elevated the RBC count, hemoglobin (Hb) %, and packed cell volume (PCV) compared to the basal diet (*p* < 0.05) (Table 5).

In this context, Nile tilapias supplemented with LEO displayed the highest response compared to the other supplemented groups (*p* < 0.05). Likewise, the WBC, heterophils, and lymphocyte counts were significantly increased with adding LEO, SP, and their combination to the diet (*p* < 0.05). Significant increases in WBC and lymphocytes were noticed in the case of LEO, SP, and their combination compared to the control group (*p* < 0.05). However, a different response of heterophils was reported. LEO, SP, and their combination markedly lowered the heterophil count (*p* < 0.05). The later response significantly lowered the H/L ratio in tilapias supplemented with LEO, SP, and their combination compared with the un-supplemented tilapias.

The serum biochemical constituents of the Nile tilapia were analyzed following LEO, SP, and their combination (Table 6).

Adding LEO and SP, separately or combined, to the Nile tilapia’s diet did not alter the biochemical profile. Only non-significant increases and decreases in TG and cholesterol levels were found, respectively. In addition, non-significant increases in the total protein and albumin concentrations were measured.

### 3.4. Histological Features of Intestine, Spleen, and Hepatopancreas of Nile Tilapia Supplemented with LEO, SP, and Their Combination

**Figure 1:**
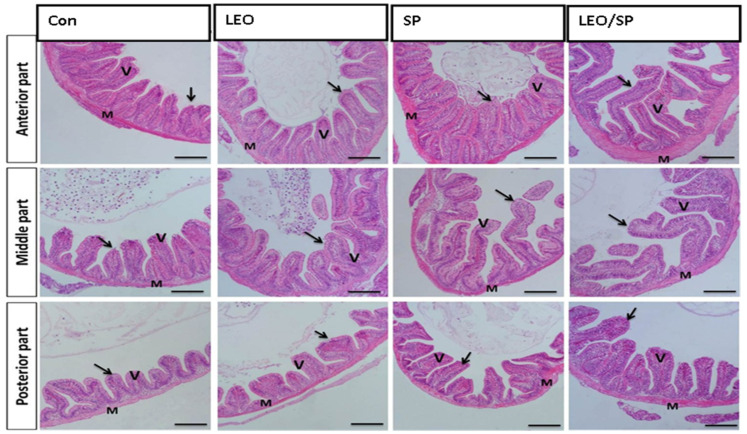
Histological features of the intestinal wall (mucosa, propria sub-mucosa, muscularis, and serosa) and intestinal villi of Nile tilapia supplemented with LEO, SP, and their combination. LEO: lemon essential oil, SP: *Spirulina platensis*. Arrow: lining enterocytes with goblet cells. V: intestinal villi. M: intestinal wall.

The histological appearance of the control fish intestine showed intact layers of the intestinal wall (mucosa, propria sub-mucosa, muscularis, and serosa) and intestinal villi (Figure 1).

The intestinal wall demonstrated a normal histomorphological appearance, where the intestinal villi and associated crypt appeared intact without any deterioration. The enterocytes lining the intestinal villi were arranged correctly. Furthermore, there was a noteworthy improvement in the construction of intestinal villi in the groups subjected to LEO and/or SP (Table 7). Accordingly, all treated groups exhibited significant increases in intestinal villi length and width and crept depth compared with the non-supplemented group (*p* < 0.05). Among the supplemented tilapia, those receiving LEO/SP consistently showed the most extensive length of intestinal villi, width, and crept depth (*p* < 0.05).

**Figure 2:**
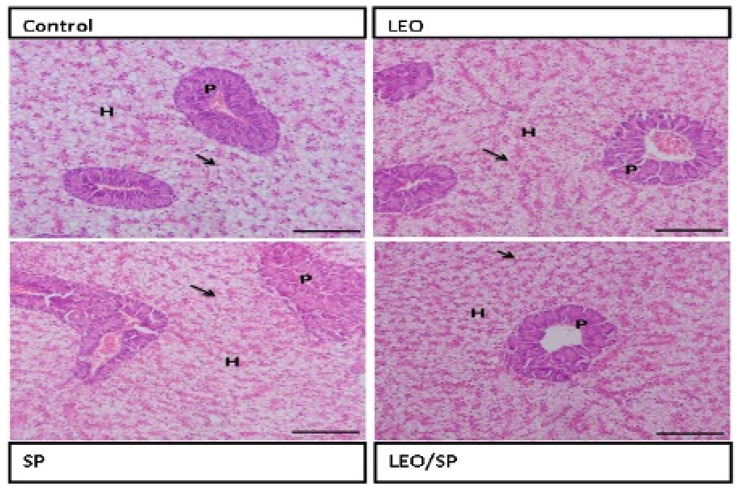
Histological features of hepatopancreas of Nile tilapia supplemented with LEO, SP, and their combination. LEO: lemon essential oil. SP: *Spirulina platensis*. H: hepatocytes. P: pancreatic acini. Arrow: glycogen deposition.

The architecture of the hepatopancreas in the control fish showed a regular appearance of hepatocytes, with large vesicular nuclei that were separated by the blood sinusoid lined by endothelial cells. LEO and/or SP supplementation improved the hepatic condition by increasing glycogen deposition within the hepatocytes, which was more evident in the group supplemented with LEO and SP. The pancreatic acini appeared normal (Figure 2).

**Figure 3:**
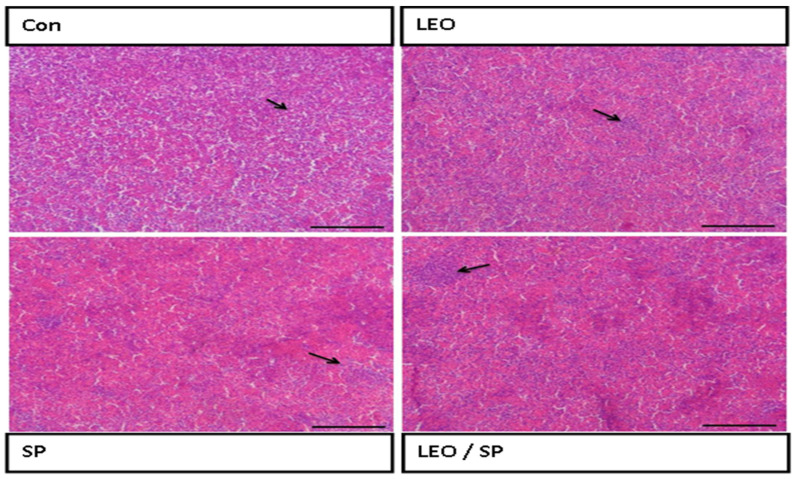
Histological features of the spleen of Nile tilapia supplemented with LEO, SP, and their combination. LEO: lemon essential oil. SP: *Spirulina platensis*. Arrow: lymphocytic aggregation in the white pulp.

The histological structure of the spleen displayed regular white and red pulps (Figure 3).

Adding LEO and/or SP to the treated groups improved the histological structure of the spleen, with increased lymphocyte infiltration (white pulp).

### 3.5. The Effects of Dietary Supplementation of LEO, SP, and Their Combination on the Expression Levels of Growth, Antioxidant, and Fat Metabolism-Regulating Genes

**Figure 4:**
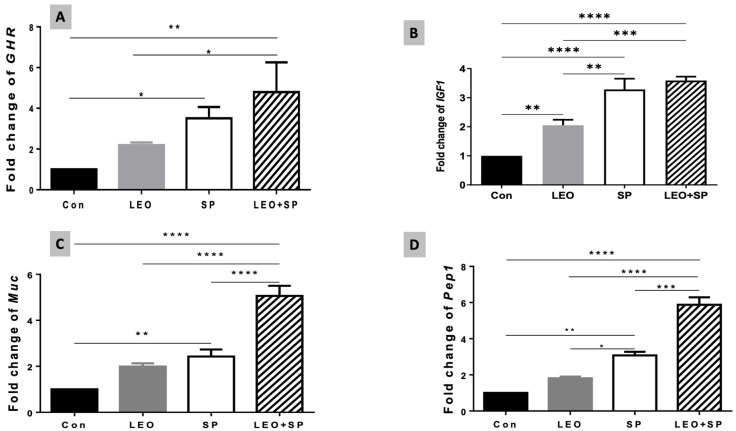
The effects of dietary supplementation of LEO, SP, and their combination on the expression levels of growth-related genes. (**A**) *Ghr1*: growth hormone receptor 1, (**B**) *Igf-1*: insulin-like growth factor 1, (**C**) *Muc*: mucin-like protein, (**D**) *Pept1*: oligo-peptide transporter 1. LEO: lemon essential oil, SP: *Spirulina platensis*. * *p* < 0.05, ** *p* < 0.01, *** *p* < 0.001, **** *p* < 0.0001, respectively.

Including both the SP and LEO/SP mixture in the Nile tilapia diet significantly upregulated mRNA levels of the *GHR* gene compared to tilapias fed the basal diet (*p* < 0.05) (Figure 4A). However, LEO supplementation did not alter *GHR* transcriptomic levels compared to the basal diet. Additionally, no marked differences were observed between the LEO and SP groups when supplemented separately, but there were significant differences between the LEO/SP mixture and LEO alone (*p* < 0.05). Likewise, adding LEO, SP, and their mixture to the Nile tilapia diet distinctly modified *IGF-1* mRNA levels (*p* < 0.05) (Figure 4B). Significant increases in *IGF-1* mRNA copies were measured in cases of LEO, SP, and their combination compared with the basal diet (*p* < 0.05). Additionally, despite being similar, the effects of either SP alone or the LEO/SP mixture were significantly higher than for LEO alone (*p* < 0.05).

The relative expression levels of mucin-like protein (*muc*) were also altered with the dietary addition of SP, LEO, and their mixture (Figure 4C, *p* < 0.05). In this regard, both SP only and the LEO/SP mixture, compared to the non-supplemented group and LEO, significantly upregulated the expression levels of the *muc* gene (*p* < 0.05). Additionally, the LEO/SP mixture induced the highest levels among all treatments and the control (*p* < 0.05).

Similarly, *Pep1* expression levels were modulated with dietary supplementation of SP, LEO, and their combination (Figure 4D, *p* < 0.05). Prominent levels of *Pep1* were found in the case of SP and the LEO/SP mixture (*p* < 0.05). Moreover, the effects of SP and the LEO/SP mixture were significantly higher than LEO and the control (*p* < 0.05).

**Figure 5:**
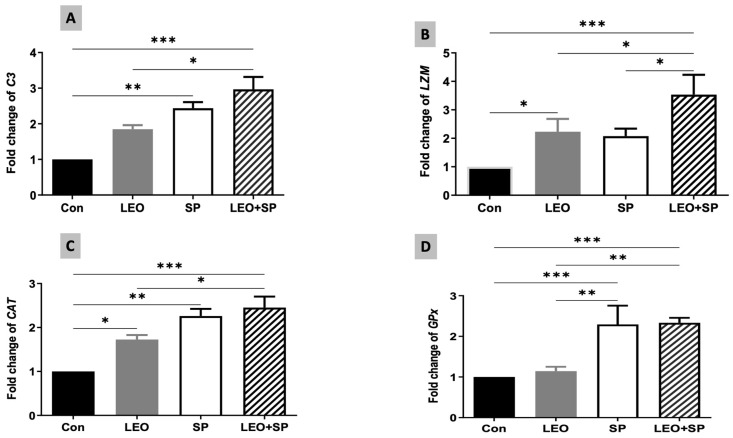
The effects of dietary supplementation of LEO, SP, and their combination on the expression levels of immune and antioxidant genes. (**A**) *C3*: complement, (**B**) *LZM*: lysozyme, (**C**) *CAT*: catalase, (**D**) *GPX*: glutathione peroxidase. LEO: lemon essential oil, SP: *Spirulina platensis*. * *p* < 0.05, ** *p* < 0.01, *** *p* < 0.001, respectively.

The expression levels of *C3* were also modified following dietary supplementation of SP, LEO, and their combination (Figure 5A). Both SP and the mixture of the two supplements distinctly increased *C3* mRNA levels compared to the LEO and the control (*p* < 0.05). Additionally, LEO alone and its mixture with SP induced significantly higher expression levels of the lysozyme gene (*LZM*) in comparison to the control group (Figure 5B, *p* < 0.05). The combination of LEO and SP displayed the highest *LZM* expression levels compared to the other treatments (*p* < 0.05).

*CAT* and *GPx* expression levels were also modified due to the dietary supplementation of SP, LEO, and their combination (Figure 5C,D). For CAT, all the supplements (SP, LEO, and their mixture) increased its expression levels compared to the un-supplemented group (*p* < 0.05). The effects of the mix were significantly higher than that of LEO alone (*p* < 0.05). Furthermore, SP and LEO/SP significantly upregulated the expression levels of *GPx* compared with LEO and the control (*p* < 0.05).

**Figure 6:**
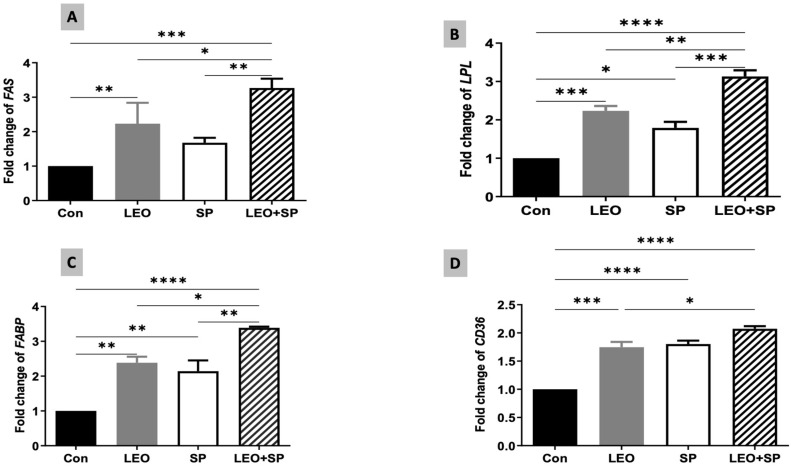
The effects of dietary supplementation of LEO, SP, and their combination on the expression levels of fat metabolism-regulating genes. (**A**) *FAS*: fatty acid synthesis, (**B**) *LPL*: lipoprotein lipase, (**C**) *FABP3*: fatty acid binding protein 3, (**D**) *CD36:* cluster of differentiation 36. LEO: lemon essential oil, SP: *Spirulina platensis*. * *p* < 0.05, ** *p* < 0.01, *** *p* < 0.001, **** *p* < 0.0001, respectively.

The relative expression levels of some fat metabolism-regulating genes were modulated by supplementing SP, LEO, and their combination in the Nile tilapia diet (Figure 6). Adding LEO and the LEO/SP mixture to the diet significantly increased the mRNA level of *FAS* compared to the control and SP (Figure 6). The LEO/SP group displayed the highest *FAS* mRNA levels among all the groups (*p* < 0.05). In addition, the *LPL* expression levels (Figure 6) were upregulated following the dietary supplementation of SP, LEO, and their mixture compared to the control group fed the basal diet (*p* < 0.05). Yet again, the effects of the LEO and SP mixture (LEO/SP) were the highest among all the groups (*p* < 0.05). Similarly, the *FABP* mRNA copies (Figure 6) significantly increased when tilapias were fed the diet supplemented with SP, LEO, and their mixture (*p* < 0.05). The LEO/SP group had the highest *FABP* mRNA copies among all the treatments (*p* < 0.05). Furthermore, mRNA expression levels of *CD36* exhibited a similar behavior because of SP, LEO, and their combination (Figure 6). Distinctly higher mRNA copies of *CD36* were found in all the supplemented groups compared to the control (*p* < 0.05). Similarly, LEO/SP had a prominent effect compared to the control and LEO group (*p* < 0.05).

## 4. Discussion

Using nutritive, non-toxic feed additives in the fish sector has gained global attention recently [54,55]. Most of the studied natural additives modulate fish performance and metabolic pathways [56,57] and enhance the fish’s immune and antioxidant status under normal and stressful conditions because of their bioactive elements [2,58,59]. These additives include phytobiotics, probiotics, and prebiotics. Combining two distinct kinds of these supplements results in more advantageous synergistic effects [4,29]. Accordingly, this study reported the most effective outcomes by combining both LEO and SP as dietary supplements, confirming their synergetic effects on Nile tilapia growth performance, immunity, and antioxidant status.

The dietary supplementation of SP (at 1%) significantly improved final body weights, body gains, SGR, and the FCR. These effects were correlated with increases in hepatic *GHR* and *IGF-1* gene transcriptomic levels. The improved growth performance in the case of SP-supplemented Nile tilapia might be linked to its highly nutritive bioactive components, such as vitamins (vitamin A and vitamin B complex) and minerals including iron, potassium, and magnesium [60]. These results agree with the findings of Rosas et al. [61], who considered SP to be one of the most recommended candidates to replace fish meal. Additionally, Nile tilapia supplemented with SP up to 15% exhibited a significantly enhanced growth rate and *Igf-1* gene expression levels [62]. Additionally, several previous studies have suggested that the dietary inclusion of a small amount of SP (1 to 5%) modifies the fish’s growth and health [35,63,64].

Nile tilapia fed LEO-supplemented diet displayed slightly enhanced BWG and SGR compared to the control group, with a decreased FCR. Moreover, LEO supplementation modulated the mRNA levels of growth-related genes; it distinctly up-regulated the *Igf-1* mRNA levels. These findings agree with the conclusions from Elsayed, Salem, and Toutou et al. [65,66], who reported that dietary supplementation of limonene from different citrus fruits could improve fish’s growth parameters and feed utilization. The beneficial effects of the essential oils (EOs) extracted from citrus fruits to aqua feed may be because of their rich contents of nutritional components [67,68]. Similarly, LEO dietary inclusion of up to 5% enhanced the growth performance indices in several fish species [19,69]. Furthermore, Nile tilapia supplemented with LEO exhibited enhanced *Igf-1* gene expression levels [12,13]. Interestingly, the best growth performance was reported for the Nile tilapias supplemented with LEO and SP, indicating their synergistic effects.

The effects of the separate dietary presence of SP or LEO on growth performance have been explored in other fish species. For example, Zhang et al. demonstrated that *Micropterus salmoides* fed different concentrations of SP showed significantly enhanced growth performance, body crude protein, muscle amino acid, and protein efficiency [70]. Moreover, common carp (*Cyprinus carpio*) supplemented with SP (30 g/kg) alone or mixed with 0.5 g/kg citric acid showed enhanced growth and immunity [71]. The supplementation of citrus lemon extract to catfish juveniles (*Pangasius hypophthalmus*) improved their growth, hematological, and innate immunity parameters and enhanced their bacterial resistance [72]. Additionally, common carp receiving 200 mg/kg of dietary limonene exhibited improved feed efficiency and increased innate immune response and resistance against *A. hydrophila* [73]. Also, limonene supplementation enhanced the antioxidant and immune response of silver catfish challenged with *A. hydrophila* as well as their hepatic histological structure and the *Igf1* mRNA levels [74]. The improved growth performance and health status due to these LEO and/or SP diets might be correlated with increased digestive enzyme levels (confirmed by increasing the expression of Muc and Pep1 genes) and improved construction of intestinal villi, which facilitates nutrient absorption and subsequently improves growth performance metrics [75,76,77].

Including LEO or SP in the Nile tilapia diet increased the mRNA levels of some fat metabolism-regulatory genes, such as *Fas*, *Lpl*, *Fabp*, and *Cd36*, with the highest expression levels reported in the case of the LEO/SP mixture. However, the LEO and/or SP combination did not alter the biochemical profile of triglycerides and only induced a slight decrease in cholesterol levels. The triglyceride concentration depends on the level of their biosynthesis and lipolysis, regulated by a series of enzymes. *LPL* is the main enzyme in triglyceride lipolysis into glycerol and free fatty acids [78]. On the other hand, fatty acid synthesis is regulated by several enzymes, such as FAS, ACC, and acyl-CoA synthetase [79].

Moreover, FA binding proteins (FABPs) and FA translocase (CD36) regulate the fatty acids’ uptake and intracellular transport [80]. In the present study, the separate supplementation of LEO or its mixture with SP did not alter serum TG levels; this could be due to increased *Lpl* gene expression. Additionally, there was an upregulation in *Fas* expression, which is unnecessary to increase the body’s triglyceride levels. FAS traditionally catalyzes the de novo synthesis of fatty acids. However, the dietary fat contents affect the regulation of the gene transcription levels and enzyme activities. The de novo synthesis of fatty acids by FAS may contribute to storing energy when the diet is rich in nutrients, especially fats and carbohydrates. However, the secreted triglycerides due to FAS appear negligible compared to other sources of fats under common dietary conditions [79]. These effects may be linked with the improved growth performance in the case of the LEO/SP combination by regulating the energy available from dietary lipids, which is one of the possible growth-stimulatory mechanisms. Accordingly, Nile tilapia supplemented with *moringa oleifera* leaves and lecithin displayed alteration in fat metabolism, as confirmed by lowered serum cholesterol and triglycerides levels with upregulated *FAS* and *LPL* mRNA levels, correlated with improved growth performance [5,50].

Previous studies have reported the essential roles of limonene and SP supplementation in fat metabolism. Accordingly, dietary limonene significantly upregulated the essential enzymes associated with *LPL* and alkaline phosphatase activities [12]. Additionally, the availability of free fatty acids significantly affects fish growth and innate immunity [81,82,83]. The effects of spirulina are perhaps due to its rich, unique composition of fatty acids and polyunsaturated fatty acids. Similarly, LDL–cholesterol was significantly decreased with SP inclusion (10%) in rainbow trout [84]. SP modulated the harmful effects of hypercholesterolemia, as it lowered the plasma cholesterol and triglyceride levels [85,86].

Intestinal health is another crucial factor regulating fish growth and feed utilization [87]. Enhancing intestinal villi integrity and density improves growth rate because they are the site of absorption and nutrient uptake [88]. Our results demonstrated that LEO and/or SP supplementation maintained the regular appearance of the intestinal wall, where the intestinal villi and associated crypts appeared intact without any deterioration. Furthermore, there was a noteworthy improvement in the construction of intestinal villi. The gastrointestinal tract’s digestive function and health correlate with mucus layer thickness [89], crucial in the intestinal tract’s innate defense and protection [90]. The mucus facilitates nutrient transportation through the gut wall [91]. Also, protein nutrient transporters, such as oligo-peptide transporter I (*Pept1*), depend on mucus as a medium for active peptide transport [92].

Goblet cell mucus secretion is controlled by mucin-like protein genes such as *Muc2* [90]. In the current study, SP or LEO supplementation significantly upregulated *Muc* and *Pep1* gene expression levels, and the highest expressions were reported in the case of the LEO and SP mixture. Several herbals improve gut nutrient digestion and utilization by upregulating mucin expression [91]. For example, orange essential oil (OEO) and LEO improved Nile tilapia growth by improving intestinal villi length, inter-villi space, and the number of goblet cells [13]. Aanyu et al. suggested that the enhanced Nile tilapia weight gain in response to limonene inclusion was due to improved protein absorption and increased mucus secretion, with significant up-regulation of *Muc* and *Pep1* genes [12]. Citrus EOs also motivated the secretion of digestive enzymes such as trypsin, amylase, amino peptidases, and alkaline phosphatase, thus improving feed utilization [93]. They also could increase the levels of beneficial gut microbes compared to pathogenic bacteria, facilitating nutrient absorption [76,94,95]. These improvements may be due to the valuable role of citrus essential oils (CEOs), which consist of some major biologically active compounds like *α*-/*β*-pinene, sabinene, *d*-limonene, *β*-myrcene, *α*-humulene, linalool, and *α*-terpineol belonging to the monoterpenes, aldehyde/alcohol, monoterpene, and sesquiterpenes group. These compounds possess anti-inflammatory, antioxidant, anticancer, and antimicrobial properties, with immense potential for food applications [96].

Similarly, SP could enhance fish growth by improving intestinal health. In this context, rainbow trout fed 5% SP showed higher intestinal villus height, absorption surface area, goblet cell numbers, and intraepithelial lymphocytes than the non-supplemented group [97]. Also, including a selenium-enriched SP diet (10%) significantly increased the number of intestinal goblet cells [98]. Nile tilapia supplemented with SP (1%) exhibited increased villi length, mucosal length, and goblet cell numbers [29]. The diet significantly increased digestive enzyme activities such as protease, amylase, and lipase, thus improving nutrient digestion and absorption [35,99].

Blood parameters could give a reliable indication of fish growth and health [100]. Fish growth, metabolic rate, and immune status affect RBC count and Hb [101]. Our results showed that SP significantly enhanced the measured hematological parameters. Moreover, adding LEO resulted in the highest RBC count, Hb%, and PCV compared to the basal diet and the other groups. Likewise, hematological parameters such as RBC, Hb, and mean corpuscular hemoglobin concentration (MCHC) were significantly increased in rainbow trout supplemented with D-limonene [14].

Moreover, the RBC count was proportionally increased by adding citrus EO up to 5% [19]. Generally, the hematological response to limonene supplementation could differ according to the source of the extract and the fish species [69,102]. However, limonene did not negatively impact fish hematology [103]. SP also improved RBC count and hematological parameters in several fish species [84,104,105].

The antioxidant response of Nile tilapia was also modified by the SP and/or LEO supplementation. Accordingly, the dietary supplementation of SP and/or LEO significantly improved the antioxidant status of Nile tilapia. They exhibited increased serum Gpx content, decreased MDA serum concentrations, and markedly improved hepatic and pancreatic histological features compared to the non-supplemented group. At the molecular level, both SP and/or LEO significantly up-regulated the expression levels of *Cat* and *Gpx* genes. Similarly, D-limonene in citrus fruit oil extract enhanced the Nile tilapia serum antioxidant enzymes, up-regulated their gene expression, and improved the liver histological features [53,106]. Also, European sea bass supplemented with *Citrus bergamia* EO, which is rich in limonene [107], showed significant increases in SOD and GPX serum concentrations [102]. D-limonene in orange peel essential oil markedly improved rainbow trout’s total myeloperoxidase and SOD serum activities and enhanced the fish survival rate against bacterial infection [14].

Additionally, limonene has a high inhibitory activity against MDA formation [108]. Likewise, spirulina dietary inclusion in catfish increased the plasma GPX concentration, lowered the MDA, and up-regulated the expression levels of *SOD*, *CAT*, and *GPX* genes after bacterial exposure [27]. Moreover, grass carp supplemented with 1% SP displayed improved antioxidant activities such as CAT and glutathione and lowered hepatic lipid peroxidation [35]. The strong antioxidant effects of SP are associated with its high contents of minerals, carotenoids, and phenolic compounds [109,110], which significantly improve the vital organ’s antioxidant activities, lower the tissue destruction level, and decrease lipid peroxidation [55,111]. Enhancing the organ’s oxidative capacity improves its histoarchitecture and delimits the destructive effects of free radicals resulting from tissue metabolism and different stressors [112,113].

The enhanced oxidative defense could indirectly activate the innate immune response through positive crosstalk of regulatory transcription factors such as Nrf2 and NF-κB, the main factors regulating the initial protective defense mechanisms [114,115]. The fish’s innate immune response is an essential non-specific defense line against pathogens and toxins via phagocytosis, lysozyme activity, and complement activity [116]. The current feeding trial demonstrated that LEO and SP enhanced PA and serum lysozyme activities. Moreover, the combined dietary supplementation of LEO and SP significantly up-regulated *lysozyme* (*lZM*) and *complement C3* gene expression levels. The synergetic effects of LEO and SP strengthened the immune response, as confirmed by the highest PA and serum lysozyme activities and the highest expression levels of *lysozyme* and *complement C3* genes. Our results agree with several previous studies that documented the potential immune-stimulating effects of the separate dietary presence of LEO and SP in several fish species. In this regard, spirulina dietary inclusion significantly motivated the plasma lysozyme activity, complement (C3), and IgM concentrations and up-regulated the mRNA levels of the *il-1β*, *il-10*, *il-8*, and *LZY* genes of the yellow catfish [34]. Spirulina supplementation at 1% also enhanced the immune response of Nile tilapia against *Pseudomonas fluorescence* infection by increasing the phagocytic and lysozyme activities and the expression levels of *IL-1β* and *TNF-α* cytokines [117]. In addition, increasing the dietary inclusion of SP in the sea bass’s diet by up to 5% boosted the fish’s immune response, as it motivated the lysozyme activity and upregulated the gene expression levels of *Il-6*, *Il-8*, *Tnf-α*, and *Tgf-β* [118].

Moreover, SP supplementation alone or mixed with *Bacillus licheniformis* enhanced the transcriptional levels of *Lysozyme*, *Il-6*, *Il-1β*, *Tgf*, and *TNF-α*, which in turn increased the goldfish resistance to bacterial infection and lowered its mortality rate [119]. Lemon EO and orange EO could efficiently enhance non-specific immunity, as they contain a high percentage of limonene. Nile tilapia diet supplemented with OEO and LEO significantly enhanced the fish’s phagocytic and lysosome activities [13]. Additionally, they showed a dose-dependent effect, where a higher dose of up to 5% dietary inclusion showed more stimulatory effects [19,102]. Enhancing the non-specific immune parameters increases fish resistance to bacterial infection and decreases mortality rates [69,120,121].

The alteration of Nile tilapia’s immune response might be correlated with altering the lipid metabolism following SP and/or LEO dietary supplementation. Fatty acids are important components in the dynamic metabolism of immune cells. They could directly or indirectly contribute to the biological processes of immunocytes, including cell proliferation and differentiation and regulating phagocytic activity [122]. The synthesis of fatty acids within immune cells is regulated by enzymes such as FAS and its related enzymes like acetoacetyl-CoA (ACC) [123]. FAS and ACC promote the cholesterol production required for toll-like receptor (TLR) signal transduction and proinflammatory macrophage activation [124]. Also, CD36 is a scavenger receptor involved in immunity and is present in mononuclear phagocytes [82]. Therefore, the increased expression levels of lipid metabolism-related genes in our study due to the LEO, SP, or LEO/SP diet would reflect the improved immune status of the fish under study. Further studies are recommended to investigate the regulatory relationship between lipid metabolism and immune-regulatory genes.

The total and the differential leucocytic counts are other important indicators of the fish’s health and immune status [125,126]. Leukocytes are the principal constituents of cellular innate immunity [127]. Many well-known dietary immunostimulants efficiently modulated the leucocytes and increased the protective lymphocyte count in the circulation [128]. Likewise, several studies showed that LEO and SP supplementation positively increased lymphocyte count [84,113]. Accordingly, our results demonstrate that the WBCs, heterophils, and lymphocyte counts were significantly modified by adding LEO, SP, and their combination to the diet, where they significantly increased WBCs and lymphocytes. However, they significantly lowered the heterophil count. Consequentially, this resulted in a significant decrease in the H/L ratio in the supplemented groups. The H/L ratio provides information about the fish’s immune status and stress conditions [129].

Based on the foremost results and discussions, this study has some limitations, including the need to study the effects of LEO, SP, and their combination under stressors during a bacterial challenge to reflect their impact on the immunity of Nile tilapia truly. This study also measured the activity of digestive enzymes using a limited sample size. Therefore, more research is advised.

## 5. Conclusions

Dietary supplementation of LEO and/or SP could improve the growth performance, feed efficiency, health status, and immune-oxidative responses of Nile tilapia. The LEO-SP mixture significantly increased the final body weight, GPX levels, PA, and WBC count. Additionally, the LEO-SP combination significantly increased the expression levels of most genes related to growth, immunity, antioxidants, and lipid metabolism. Therefore, LEO-SP could be used as a natural feed additive during aquafeed formulation to improve fish welfare through dietary management.

## Figures and Tables

**Table 1:** Composition and chemical analysis of the commercial diet used in the experiments (on a dry matter basis).

Ingredient Composition	%	Chemical Analysis	%
Fish meal (72% CP)	10	Dry matter (DM %)	93.00
Soybean meal	40	Crude protein (CP %)	30.45
Yellow corn	24	Ether extract (EE %)	7.940
Wheat bran	10	Crude fiber (CF %)	4.950
Rice bran	10	Ash %	8.660
Corn oil	3	Nitrogen-free extract (NFE %)	48.00
Dicalcium phosphate	1	Calculated energy	value
Vitamin and mineral mix	2	Gross energy (kcal/kg)	4496.36
Total	100	Metabolizable energy (Kcal/kg)	371.63

**Table 2:** Primers used in this study.

Gene	Primers	Acc. No	Ref.
*ef-1α*	F: TCAACGCTCAGGTCATCATC R: ACGGTCGATCTTCTCAACCA	XM_003458541	[49]
*β-actin*	F: CAGCAAGCAGGAGTACGATGAG R: TGTGTGGTGTGTGGTTGTTTTG	XM_003455949.2	[50]
*GHR1*	F: CAGACTTCTACGCTCAGGTC R: CTGGATTCTGAGTTGCTGTC	AY973232.1	
*IGF-1*	F: GTTTGTCTGTGGAGAGCGAGG R: GAAGCAGCACTCGTCCACG	Y10830.1	
*FABP3*	F: CAAGCCCACCACCATCATCT R: TTCCCGTCCTCTATCGTGACA	XM_003444047.5	[51]
*CD36*	F: CCCAAAGCGAACGTCACATT R: ATGTGATGCTGGAGGAAGCAA	XM_003452029.5	
*FAS*	F: TGAAACTGAAGCCTTGTGTGCC R: TCCCTGTGAGCGGAGGTGATTA	GU433188	[5]
*LPL*	F: TGCTAATGTGATTGTGGTGGAC R: GCTGATTTTGTGGTTGGTAAGG	NM_001279753.1	
*GPX*	F: CCAAGAGAACTGCAAGAACGA R: CAGGACACGTCATTCCTACAC	DQ355022	[4]
*CAT*	F: CCCAGCTCTTCATCCAGAAAC R: GCCTCCGCATTGTACTTCTT	JF801726.1	
*LZM*	F: AAGGGAAGCAGCAGCAGTTGTG R: CGTCCATGCCGTTAGCCTTGAG	XM_003460550.2	[52]
*C3*	F: GGTGTGGATGCACCTGAGAA R: GGGAAATCGGTACTTGGCCT	XM_013274267.2	
*Muc*	F: TGCCCAGGAGGTAGATATGC R: TACAGCATGAGCAGGAATGC	XM_005466350	[53]
*Pept1*	F: CAAAGCACTGGTGAAGGTCC R: CACTGCGTCAAACATGGTGA	XM_013271589	

*GHR1*: growth hormone receptor 1. *IGF-1*: insulin-like growth factor 1. *FAS*: fatty acid synthesis. *LPL*: lipoprotein lipase. *FABP3*: fatty acid binding protein 3. *CD36:* cluster of differentiation 36. *CAT*: catalase. *LZM*: lysozyme gene. *C3*: complement. *Muc*: mucin-like protein. *Pept1*: oligo-peptide transporter 1. *β-actin*: beta actin. *ef-1α*: elongation factor-1α (*ef-1α*). *GPX*: glutathione peroxidase.

**Table 3:** Growth performance of Nile tilapia after two-month dietary supplementation of bitter lemon (*Citrus limon*) peel essential oil, Spirulina, and their mixture.

	Control	LEO	SP	LEO/SP	*p*-Value
Initial wight (g)	7.73 ± 0.11 ^a^	7.88 ± 0.10 ^a^	8.08 ± 0.06 ^a^	8.07 ± 0.15 ^a^	0.124
Final weight (g)	39.50 ± 1.64 ^b^	44.67 ± 1.02 ^ab^	47.67 ± 0.42 ^a^	48.17 ± 1.21 ^a^	<0.01
Weight gain (g)	31.90 ± 1.68 ^b^	36.82 ± 1.01 ^ab^	39.58 ± 0.42 ^a^	39.77 ± 1.08 ^a^	<0.05
Feed intake (g)	60.07 ± 0.87 ^b^	61.93 ± 0.06 ^ab^	63.95 ± 0.33 ^a^	63.53 ± 0.36 ^a^	<0.01
FCR	2.03 ± 0.13 ^a^	1.68 ± 0.04 ^b^	1.63 ± 0.03 ^b^	1.61 ± 0.07 ^b^	<0.01
SGR (%/day)	1.53 ± 0.05 ^b^	1.67 ± 0.02 ^ab^	1.69 ± 0.02 ^a^	1.71 ± 0.03 ^a^	0.014
Body length (cm)	9.80 ± 0.49 ^b^	11.27 ± 0.18 ^ab^	11.52 ± 0.50 ^a^	11.60 ± 0.27 ^a^	0.012
Liver weight (g)	0.77 ± 0.09 ^a^	0.79 ± 0.07 ^a^	0.79 ± 0.07 ^a^	0.98 ± 0.12 ^a^	0.393
Intestine weight (g)	0.79 ± 0.04 ^a^	1.03 ± 0.12 ^a^	1.02 ± 0.08 ^a^	0.98 ± 0.60 ^a^	0.408
HSI (%)	1.78 ± 0.14 ^a^	1.78 ± 0.16 ^a^	1.77 ± 0.25 ^a^	2.08 ± 0.28 ^a^	0.711

FCR = feed conversion ratio; HSI = hepatosomatic index; SGR = specific growth rate. Mean values with different superscript letters within the same row significantly differ at *p* < 0.05. LEO: lemon essential oil, SP: *Spirulina platensis.*

**Table 4:** Antioxidant enzyme concentrations, phagocytic response, and lysozyme activity of Nile tilapia after two-month dietary supplementation of bitter lemon (*Citrus limon*) peel essential oil, Spirulina, and their mixture.

	Control	LEO	SP	LEO/SP	*p*-Value
SOD (IU/L)	7.95 ± 0.05 ^c^	8.75 ± 0.28 ^b^	10.07 ± 0.02 ^a^	9.96 ± 0.09 ^a^	<0.0001
GPX (IU/L)	8.39 ± 0.31 ^c^	11.05 ± 0.10 ^b^	10.78 ± 0.27 ^b^	12.39 ± 0.21 ^a^	<0.0001
MDA (IU/L)	18.54 ± 0.28 ^a^	15.55 ± 0.35 ^b^	15.31 ± 0.35 ^b^	15.11 ± 0.40 ^b^	<0.001
PA (%)	11.14 ± 0.08 ^c^	13.14 ± 0.17 ^b^	11.88 ± 0.12 ^c^	15.32 ± 0.48 ^a^	<0.0001
PI	0.98 ± 0.02	1.08 ± 0.03	1.07 ± 0.05	1.15 ± 0.038	0.1034
LZM (Unite/mL)	8.52 ± 0.31 ^c^	13.16 ± 0.22 ^a^	11.48 ± 0.19 ^b^	12.90 ± 0.11 ^a^	<0.0001

Mean values with different superscript letters within the same row significantly differ at (*p* ≤ 0.05). LEO: lemon essential oil. SP: *Spirulina platensis*. Superoxide dismutase (SOD). Glutathione peroxidase (GPX). MDA: Malondialdehyde. PA: Phagocytic activity. PI: Phagocytic activity. LZM: lysozyme activity.

**Table 5:** Hematological profile of Nile tilapia after two-month dietary supplementation of bitter lemon (*Citrus limon*) peel essential oil, Spirulina, and their mixture.

	Control	LEO	SP	LEO/SP	*p*-Values
RBCs (×10^6^/mm^3^)	3.20 ± 0.07 ^c^	3.77 ± 0.04 ^a^	3.57 ± 0.014 ^ab^	3.49 ± 0.015 ^b^	<0.001
Hb %	9.74 ± 0.15 ^c^	11.44 ± 0.07 ^a^	10.88 ± 0.06 ^b^	10.65 ± 0.06 ^b^	<0.0001
PCV (%)	31.00 ± 0.57 ^c^	37.00 ± 0.57 ^a^	34.67 ± 0.33 ^b^	34.33 ± 0.33 ^b^	<0.0001
WBCs (×10^3^/mm^3^)	10.15 ± 0.14 ^c^	11.19 ± 0.18 ^b^	11.54 ± 0.27 ^ab^	12.13 ± 0.15 ^a^	<0.001
Heterophils (%)	16.00 ± 0.57 ^a^	9.67 ± 0.33 ^b^	10.67 ± 0.33 ^b^	10.33 ± 0.33 ^b^	<0.0001
Lymphocytes (%)	74.33 ± 0.88 ^b^	82.67 ± 0.66 ^a^	80.33 ± 0.33 ^a^	80.67 ± 0.33 ^a^	<0.0001
H/L ratio	0.215 ± 0.01 ^a^	0.117 ± 0.01 ^b^	0.133 ± 0.01 ^b^	0.128 ± 0.01 ^b^	<0.0001

Mean values with different superscript letters within the same row significantly differ at (*p* ≤ 0.05). SP: *Spirulina platensis*; LEO: lemon essential oil; red blood cells (RBCs); hemoglobin (Hb); packed cell volume (PCV); white blood cells (WBCs); heterophils/lymphocytes (H/L).

**Table 6:** Biochemical analysis of Nile tilapia after two-month dietary supplementation of bitter lemon (*Citrus limon*) peel essential oil, Spirulina, and their mixture.

	Control	LEO	SP	LEO/SP	*p*-Values
TG (mg/dL)	95.47 ± 3.78	104.3 ± 3.93	105.5 ± 5.51	100.5 ± 1.42	0.325
CHOL (mg/dL)	106.3 ± 0.52	103.3 ± 2.31	102.4 ± 2.40	101.7 ± 1.89	0.254
AST (U/L)	29.59 ± 0.32	28.54 ± 1.09	28.47 ± 0.42	27.95 ± 1.03	0.553
ALT (U/L)	31.70 ± 0.75	30.59 ± 0.47	30.50 ± 0.47	29.37 ± 0.23	0.073
Glucose (mg/dL)	2.34 ± 0.09	2.68 ± 0.077	2.52 ± 0.05	2.64 ± 0.11	0.102
Albumin (g/dL)	1.51 ± 0.05	1.50 ± 0.009	1.54 ± 0.03	1.47 ± 0.04	0.729
Total protein (g/dL)	3.85 ± 0.13	4.19 ± 0.070	4.06 ± 0.04	4.12 ± 0.14	0.223

LEO: lemon essential oil; SP: Spirulina platensis; CHOL: cholesterol; TG: triglyceride; aspartate aminotransferase (AST); alanine aminotransferase (ALT).

**Table 7:** Intestinal morphometry of Nile tilapia after two-month dietary supplementation of bitter lemon (*Citrus limon*) peel essential oil, Spirulina, and their mixture.

	Items	Control	LEO	SP	LEO/SP	*p*-Value
Anterior segment	Villus height	135.6 ± 6.9 ^c^	165.3 ± 9.8 ^bc^	147.1 ± 12.8 ^b^	216.8 ± 7.5 ^a^	<0.001
	Villus width	45.11 ± 2.8 ^c^	58.81 ± 1.9 ^bc^	68.95 ± 4.1 ^ab^	82.8 ± 4.2 ^a^	<0.001
	Crypt depth	25.74 ± 1.7 ^d^	46.73 ± 4.3 ^b^	43.86 ± 1.4 ^c^	66.12 ± 1.9 ^a^	<0.0001
Middle segment	Villus height	110.3 ± 6.6 ^d^	143.3 ± 7.2 ^c^	189.6 ± 7.5 ^b^	286.9 ± 4.5 ^a^	<0.0001
	Villus width	60.6 ± 0.8 ^c^	64.30 ± 3.5 ^b^	67.7 ± 2.4 ^b^	91.4 ± 2.6 ^a^	<0.0001
	Crypt depth	31.8 ± 3.6 ^c^	40.19 ± 2.6 ^b^	42.19 ± 2.3 ^b^	65.13 ± 3.9 ^a^	<0.001
Posterior segment	Villus height	90.72 ± 3.7 ^c^	106 ± 5.4 ^c^	136.8 ± 4.8 ^b^	200.2 ± 6.1 ^a^	<0.0001
	Villus width	47.29 ± 3.9 ^c^	61.31 ± 1.7 ^b^	57.46 ± 3.9 ^bc^	77.59 ± 1.8 ^a^	<0.001
	Crypt depth	22.27 ± 0.5 ^c^	25.46 ± 0.8 ^bc^	31.11 ± 2.9 ^b^	42.77 ± 2 ^a^	<0.001

Results are expressed as means ± SE. Different lowercase letters in the row indicate statistical significance at *p* < 0.05.

## Data Availability

The data presented in this study are available from the corresponding authors upon request.
